# AI-driven transformation of precision medicine: a comprehensive narrative review of key application areas, emerging paradigms, and future directions

**DOI:** 10.3389/fpubh.2025.1656603

**Published:** 2026-01-08

**Authors:** Qin Zeng, Cheng Huang, Jun Zhu

**Affiliations:** 1 Department of Pediatric Gastroenterology Nursing, West China Second University Hospital, Sichuan University, Chengdu, Sichuan, China; 2 Key Laboratory of Birth Defects and Related Diseases of Women and Children (Sichuan University), Ministry of Education, Chengdu, Sichuan, China; 3 National Office for Maternal and Child Health Surveillance of China, National Center for Birth Defect Surveillance of China, Department of Pediatrics, West China Second University Hospital, Chengdu, Sichuan, China

**Keywords:** artificial intelligence, deep learning, healthcare ethics, personalized treatment, precision medicine, remote monitoring, symbiotic artificial intelligence

## Abstract

**Objectives:**

This study aims to elucidate the pivotal role of Artificial Intelligence (AI) in driving the transformation of precision medicine, comprehensively analyzing how it reshapes healthcare systems from traditional diagnosis and treatment paradigms into personalized health management ecosystems.

**Methods:**

A comprehensive narrative review was conducted to systematically synthesize and critically evaluate the innovative applications, paradigm shifts, and future prospects of AI across the entire precision medicine value chain. A comprehensive literature search was performed across multiple databases up to April 30, 2025, with a focus on the clinical implementation and breakthroughs of technologies such as deep learning (DL), machine learning (ML), and natural language processing (NLP).

**Results:**

AI technologies have significantly enhanced the accuracy and efficiency of disease diagnosis through medical image analysis, genomics, and multimodal data fusion. At the treatment level, AI enables the development of personalized therapeutic plans and drug dosing optimization, while revolutionarily accelerating the drug development pipeline from discovery to clinical trials. Integrated with wearable devices and telemedicine platforms, AI facilitates full-cycle health monitoring. However, the clinical translation of AI faces challenges, including an uneven evidence base, insufficient model generalizability, and ethical concerns regarding data privacy, algorithmic fairness, and interpretability.

**Conclusion:**

AI is a key driver of paradigm shift in precision medicine. To address existing challenges, future efforts should focus on generating more robust clinical evidence, adopting technologies like federated learning to ensure data privacy, and promoting the human-centered, collaborative framework of Symbiotic AI (SAI). By establishing sound ethical and governance structures, the deployment of AI technologies can be ensured to be not only efficient and advanced but also equitable and trustworthy, ultimately paving the way for an intelligent and inclusive healthcare ecosystem.

## 1 Introduction

**Figure 1:**
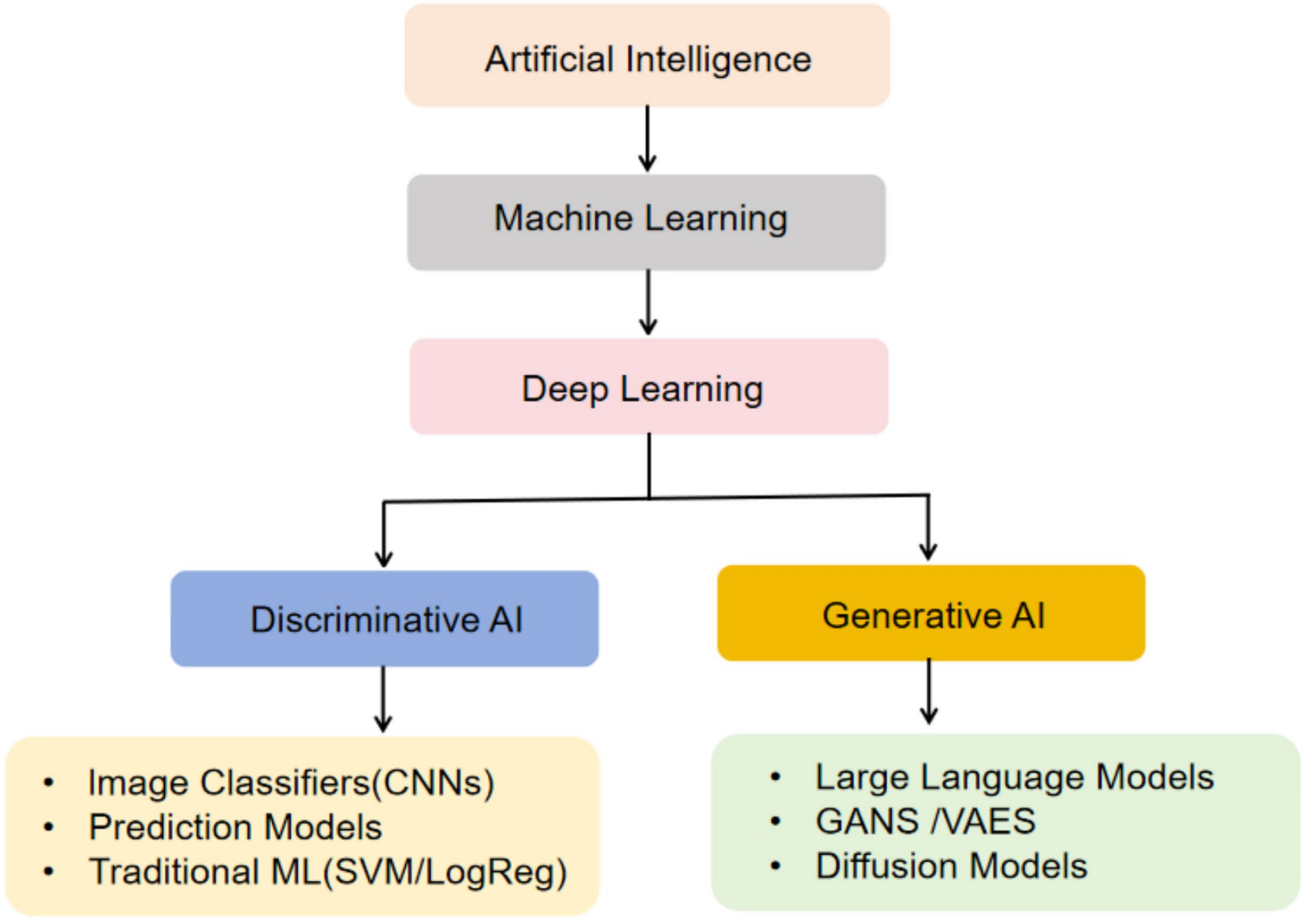
The technical hierarchy of modern artificial intelligence. This hierarchy categorizes AI based on the model’s output (discrimination vs. generation). CNNs, Convolutional Neural Networks; GANS, Generative Adversarial Networks; VAES, Variational Autoencoders; SVM, Support Vector Machine; LogReg, Logistic Regression.

In the wave of paradigm shifts in medicine, Precision Medicine is reshaping the modern medical system with its revolutionary concept of individualized diagnosis and treatment. This model integrates multidimensional data such as genomics, phenomics, and environmental exposure data (1), constructing diagnostic and therapeutic decision-making systems based on patient-specific biomarkers. The core essence lies in breaking through the limitations of traditional “population-based treatment” and achieving a paradigm shift from disease treatment to health management (2). Notably, groundbreaking advances in Artificial Intelligence (AI) are accelerating this transformation. Technologies such as imaging omics analysis driven by Deep Learning (DL) (3), clinical decision models powered by Machine Learning (ML) (4), and electronic medical record mining supported by Natural Language Processing (NLP) (5) are forming a technological matrix that drives Precision Medicine, with a hierarchy spanning from foundational ML to advanced concepts like DL and Large Language Models (LLMs) (Figure 1).

The practice of Precision Medicine essentially forms a complex data feedback loop: from genomic sequencing data and multimodal imaging data to real-time physiological parameters monitored by wearable devices, the integration and analysis of massive heterogeneous data present a significant challenge to traditional healthcare systems (6). In this context, AI technologies demonstrate unique empowering value: at the diagnostic level, convolutional neural network-based imaging recognition systems can achieve sub-millimeter lesion localization (7); in treatment decision-making, reinforcement learning (RL) algorithms can generate personalized treatment plan recommendations (8). Zhang et al. (9) systematically illustrated how AI is transforming drug development, predicting it could reduce the development cycle by up to 70%. Furthermore, AI-driven smart monitoring systems have enabled real-time dynamic monitoring of patients’ vital signs (10–12). Currently, the penetration of AI is exhibiting significant depth: the introduction of Transformer architectures has made cross-modal healthcare data fusion possible (13); the maturity of Federated Learning (FL) frameworks effectively addresses the dilemma of healthcare data silos (14); a technique that allows multiple institutions to collaborate on model training without sharing sensitive data, ensuring patient privacy (15), and intelligent consultation systems based on LLMs are reshaping doctor-patient interaction patterns (16).

However, it is important to recognize that technological evolution is always accompanied by ethical challenges—issues such as healthcare data privacy protection, lack of algorithm interpretability, and insufficient model generalization ability have become key bottlenecks restricting the deep application of AI (17–19). Excitingly, emerging technologies such as blockchain-enabled distributed learning architectures and knowledge graph-enhanced Explainable AI (XAI) offer innovative pathways to address these challenges (20–22). XAI aims to make AI decision-making transparent and interpretable for clinicians, using techniques like visual analytics and knowledge graphs to foster trust in AI-driven insights (23). Simultaneously, AI technologies demonstrate unique advantages in processing complex biological information (24). Given the interdisciplinary, heterogeneous, and rapidly evolving nature of this field, a narrative review methodology is adopted. Evidence spans multiple domains - including computational algorithms, genomics, and emerging technologies like FL - making it challenging to apply systematic reviews that require homogeneous evidence types (25). In contrast, a narrative review offers the flexibility needed to synthesize these diverse findings and analyze AI’s transformative role across the precision-medicine ecosystem.

**Figure 2:**
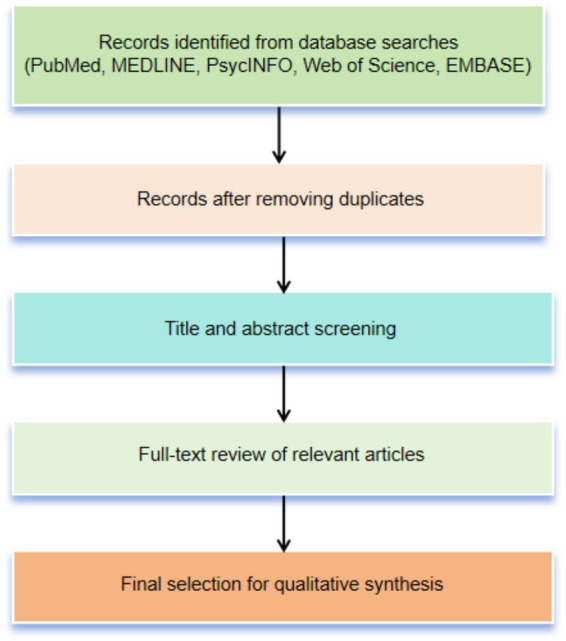
Simplified flow diagram of the literature search and selection process. This flow diagram represents an illustrative overview of the narrative review process rather than a PRISMA-based systematic workflow.

This paper systematically reviews the innovative applications of AI technologies across the entire Precision Medicine value chain. To ensure transparency, a comprehensive literature search was conducted across multiple databases, including PubMed, MEDLINE, PsycINFO, Web of Science, and EMBASE, from their inception to April 30, 2025. The search strategy focused on the intersection of “artificial intelligence” and “precision medicine,” incorporating technical keywords (deep learning, machine learning, natural language processing) and clinical terms (clinical decision support, personalized treatment, genomic biomarkers). Consistent with the narrative review methodology, literature selection was based on thematic relevance, methodological contribution, and academic impact. The initial search yielded several thousand records, which were screened to retain a few hundred core articles for full-text analysis. Inclusion criteria prioritized peer-reviewed studies directly related to AI in precision medicine, while excluding non-English and non-peer-reviewed publications. Although formal quality appraisal tools (e.g., GRADE) were not used, methodological transparency, sample robustness, and publication in high-impact journals were key selection factors to ensure the credibility and rigor of the synthesized evidence (26). A simplified flow diagram (Figure 2) visually summarizes the search and selection process.

By critically evaluating the strengths and limitations of existing technologies and incorporating cutting-edge trends, this review synthesizes a comprehensive overview of the AI-enabled technological landscape for Precision Medicine. Its primary aim is to provide theoretical support and practical reference for building an intelligent healthcare ecosystem. Furthermore, the review extends beyond technological synthesis to critically examine the varying levels of clinical validation-including performance benchmarks like AUROC and the need for more comparative studies and meta-analyses-across different medical specialties. A pivotal discussion involves the challenge of ensuring model generalizability across diverse healthcare settings, which is essential for the equitable and widespread adoption of AI in precision medicine.

## 2 Technological empowerment: core application scenarios of AI in precision medicine

AI is driving profound transformations in the fields of precision medicine and nursing. Its applications span disease diagnosis, treatment planning, drug development, and patient monitoring, providing robust support for personalized and precise medical services. To establish a technical foundation for the subsequent discussion, Table 1 provides a comparative overview of the most prominent AI techniques in precision medicine, summarizing their key applications, strengths, and limitations, serving as a quick reference guide for understanding the technologies discussed herein.

**Table 1:** Comparison of AI techniques in precision medicine.

Technique/framework	Key applications	Strengths	Limitations
Convolutional Neural Networks (CNNs)	Medical image analysis (e.g., MRI, CT scans), lesion detection, tumor classification	High accuracy in image recognition, automated feature extraction	Requires large labeled datasets, prone to overfitting, lacks interpretability
Generative Adversarial Networks (GANs)	Synthetic medical image generation, data augmentation	Enhances dataset diversity, useful for rare diseases, privacy-preserving	Quality of synthetic data may vary, ethical concerns, potential for bias
Random Forest	Genomic data analysis, disease prediction, biomarker identification	Handles high-dimensional data, interpretable, robust to overfitting	Struggles with imbalanced datasets, less effective for unstructured data
Bidirectional Encoder Representations from Transformers (BERT)	Clinical text analysis, EHR mining, patient communication analysis	Contextual understanding of text, improves NLP tasks, supports decision-making	Requires large text datasets, computationally intensive, lacks interpretability
Deep Q-Networks (DQN)	Personalized treatment optimization, dynamic treatment adjustments	Handles complex decision-making, learns from feedback, real-time adaptation	Requires high-quality real-time data, complex reward functions, suboptimal decisions possible
Multimodal Large Language Models (MLLMs)	Integration of text, images, and clinical data, personalized treatment recommendations	Holistic data analysis, improves diagnostic accuracy, supports multimodal data	Requires high-quality multimodal datasets, interpretability challenges
Explainable AI (XAI)†	Model interpretation††	Improves transparency	Potential performance trade-offs

†XAI is a framework or technique to enhance interpretability. ††The interpretability strengths refer to XAI-enhanced models; native model interpretability varies by complexity.

**Figure 3:**
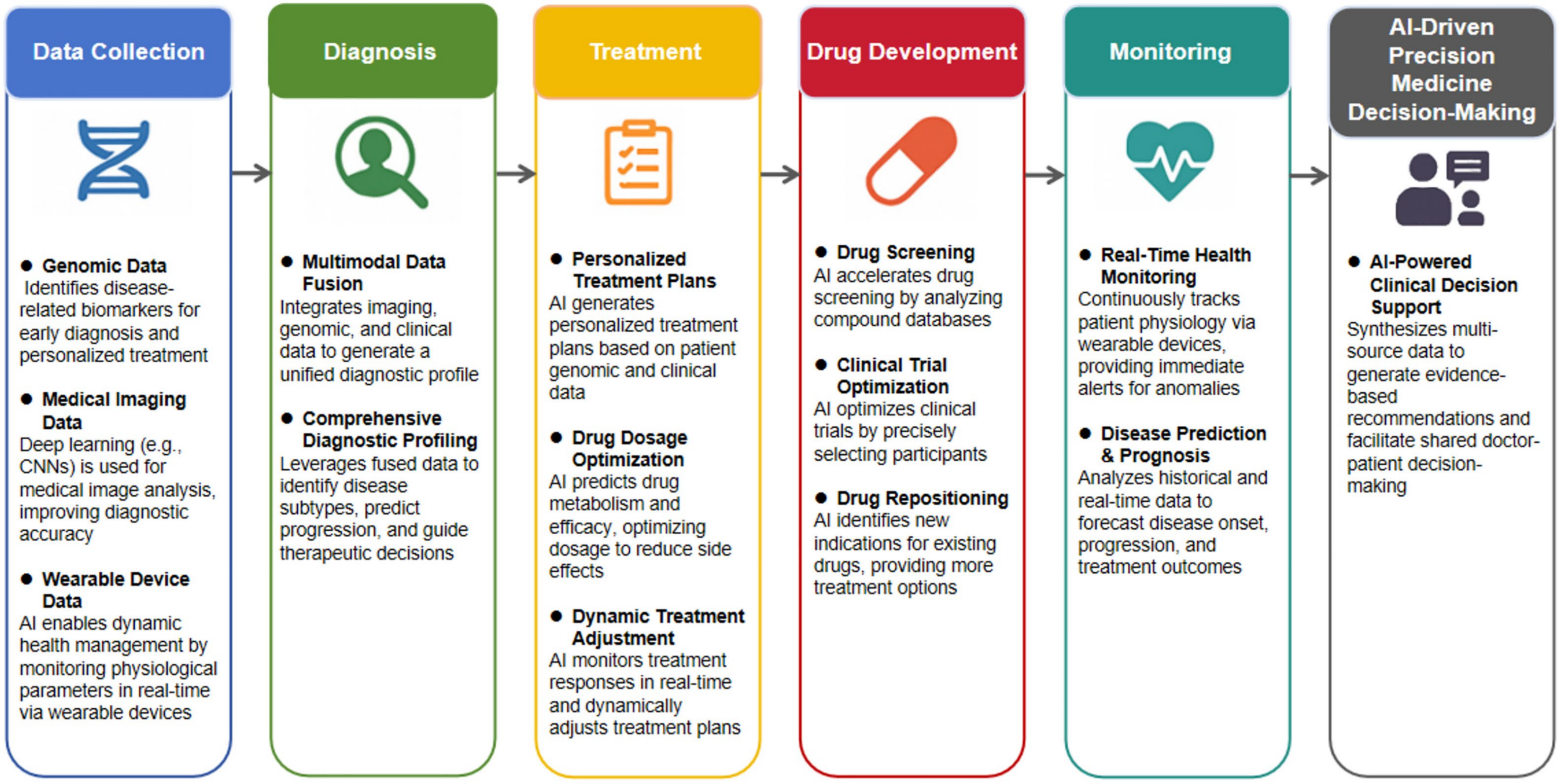
AI applications in precision medicine. This figure illustrates the workflow from data collection (genomic, imaging, wearable devices) to AI-driven decision-making, highlighting the integration of AI technologies (e.g., DL, ML, NLP) across different stages of precision medicine.

Building upon these technologies, AI demonstrates significant impact across key clinical scenarios. Before each area is explored in depth in the following subsections, Table 2 serves as a consolidated evidence map, summarizing the key findings from the literature regarding AI’s efficacy in diagnosis, treatment, drug development, and health monitoring. Furthermore, Figure 3 summarizes the diverse applications of AI across different stages of precision medicine (from data collection to diagnosis, treatment, drug development, and monitoring) from a macro-process perspective, illustrating how AI technologies are integrated to enable AI-driven decision-making through data integration, clinical decision support, and enhanced patient engagement.

**Table 2:** Synthesis of literature on AI’s role in precision medicine.

Domain	Key findings	Impact
Diagnosis	AI, particularly CNNs, improves diagnostic accuracy in medical imaging (e.g., MRI, CT scans) and lesion detection	Reduces misdiagnosis and missed diagnosis, enhances early detection of diseases like cancer
Treatment	AI optimizes personalized treatment plans by analyzing genomic data, clinical information, and lifestyle factors	Improves treatment efficacy, reduces side effects, and supports dynamic treatment adjustments
Drug development	AI accelerates drug discovery by analyzing compound databases and predicting drug activity and toxicity	Shortens development cycles and reduces costs
Health monitoring	AI, combined with wearable devices, enables real-time health monitoring and early disease prediction	Enhances proactive healthcare, reduces hospital readmissions, and improves patient outcomes
Clinical decision support	NLP and AI-driven systems extract insights from EHRs and clinical notes, supporting evidence-based decision-making	Improves diagnostic accuracy, treatment recommendations, and patient safety
Ethical challenges	AI faces challenges in data privacy, algorithmic transparency, and fairness, particularly in diverse populations	Requires robust ethical frameworks and XAI to ensure trust and equitable healthcare delivery

This table summarizes key findings from the reviewed literature, highlighting the impact of AI in precision medicine across different domains such as diagnosis, treatment, and drug development. It provides a concise overview of the evidence supporting AI’s role in transforming healthcare.

To enhance critical evaluation, this section incorporates multi-dimensional analytical perspectives when presenting each application - comparing different AI methodologies (e.g., Convolutional Neural Networks (CNNs) vs. Transformers, centralized vs. federated architectures), analyzing real-world performance limitations and underperformance cases, and addressing dataset bias and domain shift challenges - to achieve a more balanced and practical understanding of AI applications.

### 2.1 Optimization of diagnostic decision-making

This section explores how AI enhances diagnostic accuracy across data modalities, while also examining the technical and practical challenges that influence its clinical adoption.

#### 2.1.1 Medical image analysis

DL, particularly CNNs, has become a cornerstone of medical image analysis. These models learn hierarchical visual features from CT, MRI, and X-ray images to perform tasks such as disease detection, quantification, and prognosis with high accuracy (6, 27–31).

A prominent example of their impact is in mammography for breast cancer diagnosis. DL models have demonstrated performance comparable to that of professional radiologists in detecting malignancies (32–34). This capability significantly enhances diagnostic accuracy and helps reduce human errors, including misdiagnosis and missed diagnosis.

A comparative analysis reveals a trade-off between model complexity and practical applicability. While CNNs are highly effective, their performance typically depends on large, annotated datasets, and they face challenges of overfitting and interpretability in high-stakes clinical settings (35–37). More recently, Transformer-based models have shown superior ability in capturing long-range dependencies and facilitating multimodal data fusion (38, 39). However, their substantial computational cost and even greater data demands make them challenging to deploy in resource-constrained environments (40). In contrast, CNN models remain computationally more efficient and are often better suited for deployment in scenarios with limited data or computational resources, such as in primary care clinics or on portable imaging devices (23).

#### 2.1.2 Multimodal image fusion

Multimodal Large Language Models (MLLMs) align and fuse images, text, and other clinical signals via cross-modal attention to support joint reasoning for diagnosis and prognosis (13, 41); building trustworthy systems demands high-quality curated multimodal datasets and careful attention to interpretability and transparency (42–44).

AI technology can integrate data from different imaging modalities (such as MRI and CT), providing more comprehensive and three-dimensional disease information. This is particularly important in brain tumor and lung nodule diagnoses, helping clinicians accurately locate lesions, assess the nature of abnormalities, and develop more scientifically grounded treatment plans (38, 45). Recent advancements in digital pathology have further expanded the role of AI in diagnostics. For instance, AI-based systems have been successfully applied to automate the analysis of histopathological images, reducing diagnostic time and improving accuracy. A recent study demonstrated that an AI-powered platform could accurately detect, grade, and quantify prostate cancer from biopsy samples, significantly outperforming traditional diagnostic methods. This advanced system improves diagnostic accuracy and reduces interobserver variability, offering the potential to revolutionize histopathological evaluation and enhance clinical management and risk stratification for prostate cancer (46).

Nevertheless, AI models often demonstrate poor generalization when applied across hospitals or devices, a phenomenon widely documented in studies that reveal significant performance drops due to domain shift (47). This is exemplified in CT imaging, where a model developed for COVID-19 diagnosis experienced a substantial decrease in accuracy when tested on data from a different country and scanner manufacturer, directly illustrating the clinical risks of dataset bias and vendor-specific training (48).

#### 2.1.3 Genomics and biomarker identification

AI technology, particularly ML, has become instrumental in mining genomic data to extract disease-related biomarkers from large-scale datasets. By analyzing individual genetic variations, ML algorithms can predict susceptibility to specific diseases, thereby laying a crucial foundation for early diagnosis and prevention (49). For instance, Random Forest ensembles are effective at handling high-dimensional genomic features to discover disease-associated variants and build polygenic risk predictors (50, 51). However, their utility is often constrained by class imbalance and inherent interpretability challenges (50, 52).

These methods have shown significant promise in complex disorders. A notable example is the application of AI models to identify genetic markers for Alzheimer’s disease, facilitating early intervention strategies. Miller et al. (53) recently demonstrated the potential of such approaches in Alzheimer’s genetics, underscoring their role in discovering novel markers and predicting individual risk.

A critical limitation, however, lies in the generalization of these models across diverse populations. The heterogeneity and high dimensionality of genomic data mean that AI models often face significant performance degradation when applied across different ethnic groups. Such algorithmic bias, where models trained on non-representative data fail to generalize, can exacerbate health disparities, as demonstrated in studies of clinical decision-making algorithms (54). In genomics, this is starkly evident in polygenic risk scores, where models trained predominantly on European-ancestry data show markedly reduced predictive accuracy in non-European populations, ultimately impacting the fairness and accuracy of early disease identification (55).

#### 2.1.4 Comprehensive clinical data analysis

The application of AI in precise diagnostics extends beyond imaging and genomics to encompass the comprehensive analysis of clinical data, laboratory results, and lifestyle information, thereby supporting more accurate individualized diagnoses. Specifically, Transformer-based clinical NLP models, such as Bidirectional Encoder Representations from Transformers (BERT) variants, excel at encoding contextual semantics within Electronic Health Records (EHRs) to extract phenotypes, medications, and clinical outcomes for downstream modeling and cohort construction (56–58).

These technologies have yielded significant real-world impact. For instance, by analyzing EHR data, AI can identify high-risk patients and enable early intervention, effectively reducing the incidence of severe diseases (59, 60). In the field of telemedicine, AI-driven diagnostic tools integrated into healthcare platforms provide real-time diagnosis and consultation for patients in underserved areas, effectively bridging geographical disparities in healthcare access (61).

However, reveals both the considerable promise and the accompanying challenges of these applications. The core strength lies in the capability to process vast, multi-source clinical data to enhance diagnostic accuracy and decision-making efficiency. Nevertheless, the effectiveness of these advanced models is highly dependent on large-scale, domain-specific corpora and substantial computational resources (57). More critically, the equitable deployment of AI faces systemic challenges. Algorithmic biases can perpetuate and amplify existing health disparities, as evidenced by studies on population health management algorithms (54). Concurrently, persistent digital literacy and technology access gaps between different regions and populations risk excluding the most vulnerable from the benefits of innovation (62). Proactively addressing these inequities is a critical prerequisite for the responsible and effective future deployment of AI in healthcare.

#### 2.1.5 Dynamic monitoring and dis ease prediction

AI plays a crucial role in dynamic monitoring and disease prediction by continuously analyzing changes in patients’ vital signs and biomarkers to support clinical decision-making (30). Technically, RL algorithms such as Deep Q-Networks (DQN) learn adaptive alerting or treatment policies from longitudinal vital-sign and laboratory data streams by optimizing long-term rewards (63, 64).

A compelling application of this approach is demonstrated in cardiovascular disease management. Hinrichs et al. (65), for instance, conducted an analysis of data from the TIM-HF2 trial, which incorporated daily transmissions from non-invasive monitoring devices. They developed a machine learning model to predict the risk of heart failure hospitalization within seven days. The AI model reliably identified high-risk patients requiring immediate intervention, outperforming a conventional heuristic algorithm in terms of the area under the ROC curve and significantly reducing the need for manual data review.

A comparative analysis, however, highlights both the promise and the persisting challenges. While AI demonstrates significant potential in dynamic forecasting, its effectiveness is constrained by several factors. Firstly, model performance hinges on high-quality, real-time data streams and carefully specified reward signals (63). Secondly, akin to many complex AI systems, the transparency and interpretability of the model’s decision-making process remain a major barrier to its widespread adoption and trust in clinical practice (44). Addressing these twin challenges of data quality and model interpretability is pivotal for advancing the field toward mature clinical application.

In summary, AI applications are revolutionizing disease diagnosis. The transition from precise diagnostic insights to personalized therapeutic actions forms the foundation of a closed-loop precision medicine system, which is the focus of the next section on treatment management.

### 2.2 Treatment management in a closed loop

This section explores how AI leverages diagnostic insights to formulate, execute, and dynamically adjust personalized treatment strategies, thereby establishing an optimized closed-loop system for clinical intervention.

#### 2.2.1 Personalized treatment plans

In precision medicine, imaging CNNs, genomic Random Forests, and RL/DQN policies are increasingly integrated for sequential decision-making and therapy adaptation (50, 51, 63, 64).

AI’s application in personalized treatment plan formulation is particularly prominent, as it leverages ML technologies to identify key biomarkers from a patient’s genetic information, predict drug responses, and provide individualized treatment decision support for clinicians (66). Multiple studies show that AI models have broad potential in oncology. For example, Rakaee et al. (67) developed and evaluated a DL model to predict the response of advanced non-small cell lung cancer (NSCLC) patients to immune checkpoint inhibitors (ICI), achieving high prediction accuracy. Similarly, Du et al. (68) developed an MRI-based self-attention network (MESN) to predict pathological complete response (pCR) in breast cancer patients, which outperformed traditional models (69). IBM Watson for Oncology is a notable example, providing evidence-based recommendations based on a patient’s specific molecular profile (70).

Despite its potential, ensuring the generalizability and validation of these models across diverse populations remains a critical challenge.

#### 2.2.2 Drug dosing optimization

Pharmacogenomic ML predicts patient response to drugs, while RL-style control adjusts dosing in real-time using streaming data from labs and vital signs (63, 64, 71).

Building on personalized treatment plans, AI plays a key role in the individualization of drug dosing. By analyzing a patient’s physiological characteristics and genetic background, AI can predict the rate of drug metabolism and efficacy, allowing for the development of personalized drug dosages that improve efficacy and reduce side effects (72). For example, AI has been used in genetic testing for warfarin dosing to predict optimal doses based on individual genetic markers (73). Furthermore, AI systems have the capability to dynamically adjust treatment plans, continuously monitoring patients’ treatment responses and modifying plans accordingly. This ability is especially critical in the management of chronic diseases (74). Technologies such as AI-driven telemedicine platforms, which enable real-time monitoring and management, are increasingly being integrated into healthcare settings (75). Nevertheless, challenges related to data privacy, real-time data quality, and system interoperability must be overcome. Concerns regarding the accessibility and applicability of genetic testing in resource-limited settings also remain a significant barrier.

#### 2.2.3 AI-assisted surgery and robotics

In robotic and image-guided procedures, computer-vision DL models provide perception (segmentation, tracking) and decision support for guidance and safety (6, 30).

Beyond drug dosing and treatment planning, emerging technologies such as AI-assisted surgery and robotics are transforming treatment management. For example, Intuitive Surgical’s da Vinci system, a robotic surgery platform powered by AI, enables more precise and minimally invasive procedures (76). In neuro-rehabilitation, AI systems are being used to personalize rehabilitation programs, adjusting therapeutic interventions based on real-time patient data, improving outcomes in stroke recovery (77). On the other hand, these technologies require further development to overcome regulatory and technical challenges.

AI, through deep analysis of individual data, aids in creating personalized treatment plans, optimizing therapeutic effects, and reducing side effects. Nevertheless, challenges related to data integration, privacy, and the development of universally applicable AI models need to be addressed.

### 2.3 Innovation in drug development

This section examines how AI is accelerating and optimizing various stages of the drug development pipeline, from initial discovery to clinical deployment.

#### 2.3.1 Drug discovery and personalized design

DL models learn structure–activity relationships and protein/ligand representations, while GANs augment scarce assay or imaging data to improve robustness during candidate discovery (78–81).

AI occupies a central role in drug development. By leveraging DL and ML technologies, AI can analyze vast compound databases, identify potential drug candidates, and predict their activity and toxicity, significantly accelerating the drug screening process (82). For example, AlphaFold, a breakthrough AI model, has provided unprecedented insights into protein structures, which are essential for drug target identification (83). Additionally, by combining patient genetic and biomarker information, AI assists in the design of personalized treatment plans, improving drug efficacy and reducing adverse reactions (84). IBM Watson for Drug Discovery is a notable example, integrating patient data to accelerate the development of personalized cancer therapies (85).

However, challenges such as the reliability of AI predictions in real-world populations require further validation. The integration of multi-modal data (genetic, clinical, environmental) is still in its infancy, and data privacy and ethical concerns remain major barriers.

#### 2.3.2 Clinical trial optimization

NLP (e.g., BERT) parses eligibility criteria and outcomes from protocols and EHRs, while RL supports adaptive randomization and simulation for trial optimization (57, 64).

Building on the advancements in drug discovery, AI contributes to optimizing clinical trial design by precisely selecting trial participants, improving trial efficiency, and predicting trial results. For instance, Manz et al. (86) developed an AI-powered clinical trial simulation tool to predict outcomes and improve recruitment strategies, reducing trial timelines and ensuring that participants most likely to benefit from the treatment are included (87). A key challenge is the generalizability of AI models across different demographics and regions, which needs further exploration.

#### 2.3.3 Drug repositioning and interaction prediction

Literature - and omics - driven repositioning blends clinical NLP with ML representations to propose new indications for existing drugs (56, 57). Similarly, clinical NLP and sequence/graph ML extract medication entities and relations from EHRs and biomedical text to surface plausible drug–drug interactions (56, 57).

Beyond new drug development, AI shows remarkable advantages in drug repositioning, enabling the discovery of new indications for existing drugs, thereby offering patients more treatment options (88). For example, Deep Repositioning has been used to identify novel applications of approved drugs for rare diseases (89). In addition, AI can predict drug interactions, assisting clinicians in developing safe and rational drug regimens, particularly for patients requiring polypharmacy (74). By analyzing large clinical databases, AI can detect potential interactions. IBM Watson for Oncology has been extended to predict drug interactions in cancer treatment regimens, with a recent study demonstrating enhanced capability by integrating a knowledge graph, building upon its core purpose of providing evidence-based recommendations and aligning with data-driven drug safety profiling (70).

Nevertheless, there is still a need for validation in clinical settings for both repositioned drugs and predicted interactions, as the potential for side effects must be carefully considered. AI systems must be further developed to incorporate real-world clinical data and provide actionable, clinically relevant results.

#### 2.3.4 Emerging technologies in drug development

Generative models (e.g., GANs) and reinforcement learning can simulate patient trajectories and augment sparse datasets to inform digital-twin-style exploration and delivery strategies (64, 78, 80).

Looking to the future, emerging technologies such as digital twins are increasingly shaping drug development. Digital twins, which create virtual representations of patients, can simulate how a patient might respond to different drugs, offering real-time feedback for drug development and personalized treatment (90). Similarly, AI-assisted robotics enhance the precision of drug delivery and treatment, particularly in fields like orthopedics and neurology (91–93).

That said, integrating these technologies into drug development pipelines requires overcoming significant regulatory and technical challenges.

Overall, AI enhances the drug development process across multiple stages, effectively improving development efficiency and success rates while providing more accurate treatment options for patients. However, challenges related to data validation, ethical considerations, and the integration of emerging technologies need to be addressed.

### 2.4 Full-cycle health monitoring

This section outlines the role of AI and connected devices in enabling continuous, personalized health management outside traditional clinical settings.

#### 2.4.1 Real-time monitoring with wearable devices

On-device DNNs denoise and compress biosignals, while RL/DQN and time-series models track longitudinal risk and recommend just-in-time actions (6, 63, 64, 94).

AI, combined with wearable devices (e.g., smartwatches, biosensors), shows immense potential in health management. These devices continuously monitor users’ physiological parameters, creating individualized health profiles. AI analyzes these data to assess potential health risks, such as evaluating cardiovascular health status through heart rate variability (95). A notable example is the Heart Study, which employs AI algorithms to identify cardiac arrhythmias through smartwatch-derived data (96).

Nevertheless, challenges remain in data standardization, data privacy, and the accuracy of AI predictions in diverse populations.

#### 2.4.2 Disease prediction and early warning

Sequential DL and RL frameworks forecast short-term physiological trends from continuous data streams (63, 64).

Building on real-time monitoring, AI technology uses historical data and ML algorithms to predict disease onset trends. For diabetic patients, AI can predict blood sugar fluctuations based on blood sugar levels, diet, and activity, issuing early warnings and guiding preventive measures (97). Diabetes Watch is a notable example, analyzing real-time data to provide insights and recommendations for managing diabetes (98). On the other hand, issues related to data quality and the need for large, diverse datasets for accurate predictions persist.

#### 2.4.3 Real-time alerts and personalized management

Signal-processing with CNNs plus policy learning enables real-time anomaly detection and triage from wearable and bedside monitors (6, 64). NLP-driven coaching systems and RL personalize lifestyle recommendations and education using longitudinal data (58, 64, 99).

AI achieves real-time monitoring of patients’ health status, automatically pushing warnings or interventions when necessary (e.g., for abnormal heart rate patterns) (100). Research on wearable biosensors indicates promising approaches for non-invasive biomarker detection (101). Moreover, AI facilitates personalized health management, offering tailored lifestyle recommendations (e.g., personalized exercise and diet plans) based on individual health data (102). The Cardiogram app, for instance, uses AI to analyze heart rate data and offers personalized lifestyle recommendations (103).

However, issues such as data accuracy, system integration with existing healthcare infrastructures, and the management of false positives remain critical. Concerns about the accessibility of wearable devices and ensuring equitable access to AI-powered health management solutions, especially in low-resource settings, also remain.

#### 2.4.4 Integration with telemedicine platforms

Multimodal models fuse wearable streams with clinical text and images to summarize patient state for remote decision support (13, 41). By embedding these fusion models into telemedicine backends, clinicians can receive push-style, evidence-graded summaries without manual data review.

The practical value of this integration is demonstrated by research into AI-powered remote monitoring systems. These systems synthesize real-time data from wearable devices (e.g., for monitoring heart failure or chronic obstructive pulmonary disease) with patients’ EHRs (65, 95). By applying predictive analytics, they can identify patients at high risk of clinical deterioration, enabling timely intervention by healthcare providers. Studies have shown that such integrated approaches can reduce hospital readmission rates for patients with chronic conditions by facilitating proactive and personalized care management (65, 95).

The advancement of these integrated platforms, however, underscores persistent challenges. Key issues that must be addressed include achieving robust cross-platform data interoperability, ensuring the security of longitudinal data across the care continuum, and conducting rigorous validation of AI-driven clinical summaries across diverse healthcare settings and patient populations to ensure their reliability and generalizability.

## 3 Paradigm innovation: AI-driven transformation of the healthcare system

**Figure 4:**
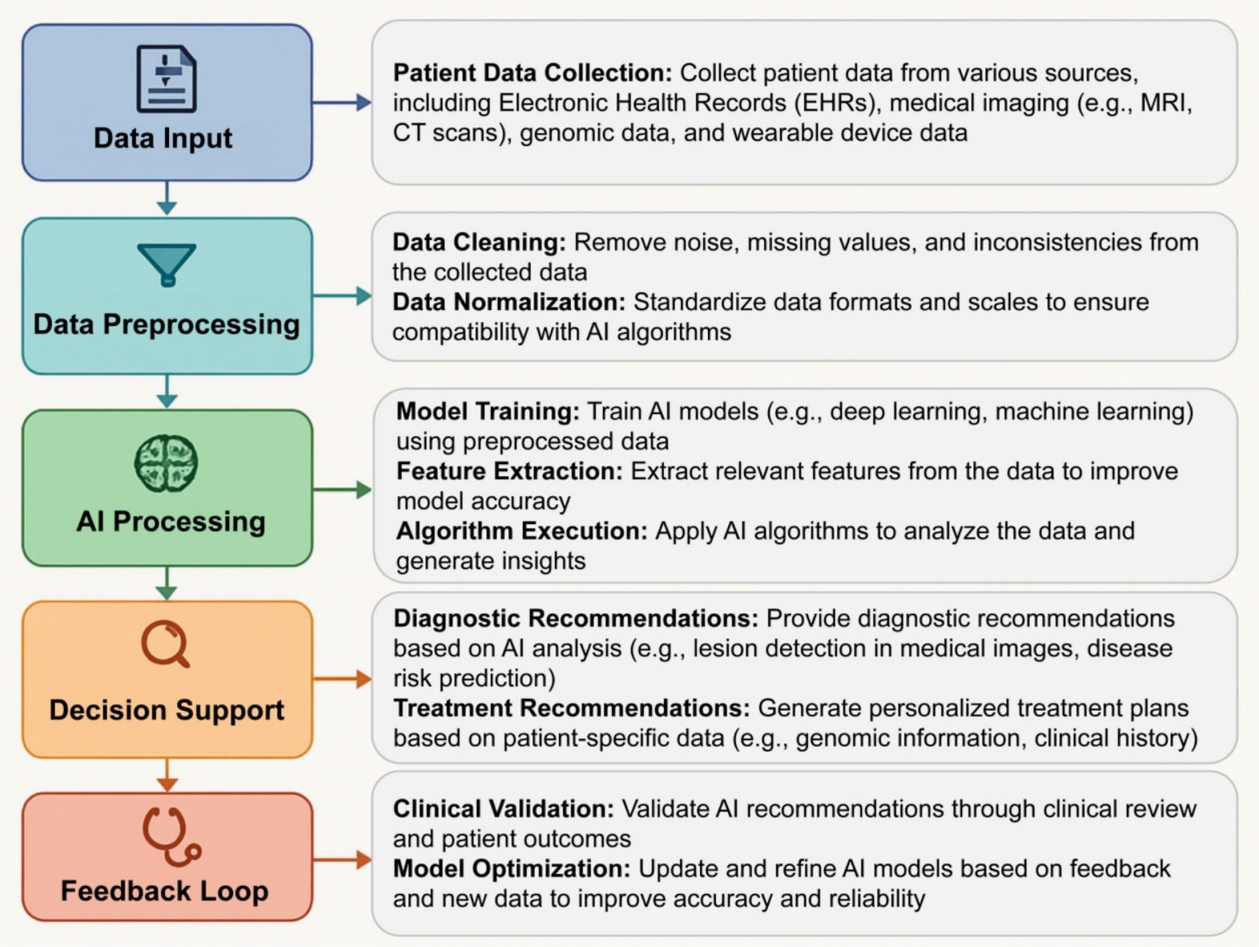
AI algorithm operational flow in clinical settings. This figure illustrates the workflow from data input (e.g., EHRs, medical imaging) to decision support and feedback loops, highlighting the integration of AI technologies in clinical settings.

AI applications in precision medicine and nursing are driving a significant transformation in healthcare systems, transitioning them from reactive to proactive care. This shift involves not only personalized healthcare, remote monitoring, and intelligent decision support systems but also improvements in hospital logistics, patient flow, and administrative efficiency. Figure 4 illustrates the operational flow of AI algorithms in clinical settings, showing how patient data is processed to provide diagnostic and treatment recommendations, and how feedback loops optimize AI models. This figure highlights the key stages of AI integration, from data input to decision support and model optimization, providing a comprehensive view of AI’s role in reshaping healthcare workflows. The following subsections delve into the specific ways AI is transforming service models, system capabilities, and the healthcare ecosystem.

### 3.1 Service model reconstruction

Personalized medicine is a key component of AI-driven transformation. AI tailors treatment plans by integrating individual genetic information, biomarkers, and lifestyle factors, enabling more effective and customized treatments. For example, AI systems have been used in oncology to analyze genomic data, identifying specific tumor mutations and helping clinicians select the most effective targeted therapies for cancer patients (104). Additionally, AI can predict patient responses to drugs and optimize dosage settings to minimize adverse reactions (105). AI-driven tools such as IBM Watson for Oncology use AI to analyze patient data, including genetic profiles, to suggest personalized treatment options based on the latest research and clinical data, significantly improving the speed and accuracy of treatment plans (106).

Moreover, AI is being used to predict and monitor chronic conditions, leading to a shift from traditional reactive care to proactive, preventive care. For instance, AI plays a role in managing diabetes by predicting blood sugar fluctuations and adjusting insulin doses automatically, thereby reducing the risk of emergencies and improving long-term patient health (107).

### 3.2 System capability upgrades

AI has fundamentally altered healthcare through the development of remote monitoring and predictive analytics, greatly enhancing both the efficiency of patient care and the allocation of medical resources. For example, AI-powered systems such as remote ECG monitoring platforms use wearable devices to collect patient data in real-time. These systems can analyze heart rate, rhythm, and activity levels to predict potential heart attacks or strokes before they occur (108). In practical applications, Li et al.’s (109) research shows that wearable devices integrated with AI algorithms continuously monitor patients and alert clinicians to early signs of deterioration, reducing hospital readmission rates and minimizing unnecessary emergency visits.

Furthermore, AI’s integration with IoT devices supports seamless, dynamic health management, particularly for older adult patients with chronic conditions (110). AI models predict health deterioration in real time, enabling timely intervention and reducing hospital congestion (111). By enabling continuous health assessments, AI in remote monitoring not only improves the patient’s quality of life but also reduces the strain on hospitals and clinics, particularly in managing large patient populations in resource-limited settings (112).

### 3.3 Ecosystem evolution

Intelligent Decision Support Systems (IDSS) are increasingly used to process vast amounts of medical data, helping healthcare professionals make faster and more accurate decisions (113). For example, studies have shown that AI models deployed in radiology departments assist clinicians by identifying patterns in medical images such as CT scans and MRIs, thereby improving diagnostic accuracy and treatment outcomes (114). These systems are also integrated with EHR, enabling personalized treatment recommendations based on patient characteristics and medical history (114).

AI-driven systems are optimizing hospital logistics and patient flow by predicting patient demand, improving resource allocation, and reducing wait times. For instance, AI algorithms predict emergency department admission rates, helping hospital managers allocate resources more efficiently and avoid bottlenecks (115). This proactive management of patient flow minimizes overcrowding in hospitals, reduces patient waiting times, and optimizes bed usage, leading to better patient experiences and improved operational efficiency (116).

Additionally, AI is significantly enhancing administrative efficiency by automating routine tasks such as scheduling, billing, and documentation (117, 118). For example, AI-powered administrative assistants are now helping hospitals streamline patient scheduling by predicting the most optimal times for appointments and automatically adjusting to cancelations or no-shows, thereby reducing administrative overhead and improving service availability (119).

In conclusion, the integration of AI in precision medicine, remote monitoring, and intelligent decision support systems is driving a shift from reactive to proactive care in healthcare. Real-world applications such as AI-driven personalized cancer treatments, predictive health monitoring using IoT, and automated administrative processes are fundamentally transforming how healthcare is delivered. AI is enhancing hospital logistics, patient flow, and resource allocation, leading to improved hospital operations and greater overall efficiency. As technology continues to evolve, AI will play an increasingly pivotal role in providing precise, proactive, and personalized healthcare experiences, optimizing both clinical outcomes and operational efficiency.

## 4 Development challenges and future prospects

Table 3 (Summary of Development Challenges and Future Prospects in AI Healthcare) outlines the key ethical, privacy, and governance challenges in AI healthcare implementation, along with their impacts and potential future directions. Addressing these issues is crucial for ensuring the responsible and equitable deployment of AI technologies in healthcare. By integrating robust ethical frameworks, enhancing algorithmic transparency, and fostering cross-institutional collaboration, we can mitigate the risks associated with AI deployment while maximizing its potential to transform healthcare delivery. The future of AI in healthcare lies in its ability to align with human values, ensuring equitable access and trust among stakeholders.

**Table 3:** Summary of development challenges and future prospects in AI healthcare.

Domain	Key challenges	Impact	Future directions
Ethical challenges	Data privacy, algorithmic transparency, fairness, and bias in AI models	Hinders trust, equitable healthcare delivery, and adoption of AI systems	Implement XAI, federated learning, and fairness protocols; ensure diverse datasets
Privacy constraints	Data sharing barriers, regulatory compliance (e.g., HIPAA, GDPR), and data leaks	Limits AI development and cross-institutional collaboration	Use secure data-sharing methods (e.g., blockchain) and federated learning
Governance and accountability	Lack of clear liability frameworks for AI errors	Delays AI adoption and raises ethical concerns	Develop standardized governance structures and define roles for AI developers and practitioners
Algorithmic bias	Biased datasets leading to unequal outcomes for diverse populations	Perpetuates healthcare disparities and reduces AI reliability	Use data diversification, fairness auditing, and algorithmic transparency tools
Clinical limitations	AI tools may fail to generalize in real-world settings; over-reliance on AI	Erosion of clinical skills and potential patient harm	Continuous validation, human oversight, and training for healthcare professionals
Regulatory challenges	Varying regional regulations, long approval timelines, and lack of standardization	Hinders multinational AI adoption and integration	Develop international AI regulatory frameworks and clear certification guidelines
Institutional resistance	Concerns about job displacement, lack of infrastructure, and trust in AI	Slows AI adoption and integration into healthcare systems	Invest in training programs, infrastructure, and foster trust in AI’s role in care
Symbiotic AI (SAI)	Not applicable	Not applicable	Develop tools integrating SAl, using XAl and FL to advance healthcare Al

XAI, Explainable Artificial Intelligence; HIPAA, Health Insurance Portability and Accountability Act; GDPR, General Data Protection Regulation; SAI, Symbiotic Artificial Intelligence; FL, federated learning.

### 4.1 Heterogeneity of clinical evidence and generalizability challenges

A critical synthesis of the literature presented in this review reveals significant disparities in the maturity of clinical evidence supporting AI applications across different medical specialties. In well-established domains such as oncology (e.g., breast cancer screening with mammography) and cardiology (e.g., echocardiogram interpretation), AI models have been validated in larger, often multi-center studies. Crucially, a growing body of comparative studies and meta-analyses in these fields now provides evidence for benchmarking AI performance directly against conventional standard care. For specific, narrow tasks, some deep learning models demonstrate performance on par with or even surpassing that of human experts, with reported metrics frequently achieving AUROC ranges of 0.89 to 0.90 (94, 120–122). However, it is essential to note that this technical equivalence or superiority in controlled studies does not automatically translate to superior patient outcomes in routine practice, and evidence demonstrating a clear improvement in final patient endpoints over standard care remains less common.

Conversely, as this review identifies, areas such as infectious disease, maternal-fetal medicine, and pediatrics remain relatively nascent. The evidence here is often derived from smaller, single-center, retrospective cohorts, making direct comparisons with the efficacy of established standard care approaches difficult and highlighting an urgent need for more robust external validation and prospective trials (123).

Beyond the variability in evidence across specialties, a paramount and cross-cutting challenge is the generalizability of AI models between high-resource and low-resource settings. Models excelling in data-rich environments often suffer from performance degradation due to “domain shift” when faced with different patient demographics, imaging equipment, or clinical practices in resource-constrained settings (124). For instance, a landmark comparative study by Zech et al. (47) demonstrated that a chest X-ray model trained on data from one US hospital system experienced a significant drop in performance when applied to data from other hospitals within the same network, thereby failing to generalize its initially promising performance versus local diagnostic standards. This underscores that an AI model’s performance cannot be evaluated in isolation but must be proven robust and superior to relevant local standard care across diverse environments.

Addressing these challenges requires a dual approach: first, the promotion of rigorous, multi-center clinical trials designed specifically to compare AI-integrated care pathways against current standard of care and the synthesis of existing evidence through systematic reviews and meta-analyses to establish robust performance benchmarks; and second, the technical development and adoption of strategies like federated learning for privacy-preserving multi-institutional collaboration and domain adaptation methods to enhance model robustness across diverse clinical environments.

### 4.2 Ethical challenges in AI implementation

The deployment of AI in healthcare brings forth significant ethical and privacy constraints that can impede its widespread use. Ethical concerns regarding data privacy, algorithm transparency, and fairness must be carefully addressed to build trust and ensure equitable healthcare delivery. Data privacy remains one of the most significant ethical challenges in AI implementation. Healthcare data, including sensitive patient information, must be handled with the utmost care to protect privacy and comply with regulations such as the Health Insurance Portability and Accountability Act (HIPAA) in the United States or the General Data Protection Regulation (GDPR) in Europe (125, 126). However, despite the benefits of data sharing in AI development, these regulations often create friction, as sharing high-quality data across institutions becomes complicated due to privacy concerns (127). Even though solutions such as FL can mitigate privacy risks by allowing data to stay localized, they are still not sufficient in addressing the real-world complexities of cross-institutional collaboration (128). Moreover, the challenge of ensuring secure data transmission and preventing breaches remains a constant concern, as healthcare organizations continue to grapple with high-profile data leaks (129).

Another key ethical issue in AI deployment is the lack of algorithmic transparency. AI models are often considered “black boxes” in healthcare, meaning their decision-making processes are not easily understood by healthcare professionals (130). This lack of interpretability raises concerns about the trustworthiness of AI systems in critical healthcare decisions. If healthcare professionals cannot fully understand the rationale behind AI recommendations, they may hesitate to rely on them, potentially delaying or undermining patient care (131, 132). To address these concerns, it is essential that future AI models incorporate XAI techniques such as knowledge graphs, causal inference, and visual analytics (133, 134). These methods will not only help clarify the decision-making process but also foster collaboration between AI systems and healthcare professionals, ensuring that AI is viewed as a tool to support—not replace—human judgment.

Furthermore, the issue of fairness in AI remains a crucial challenge. AI systems are often trained on datasets that lack diversity, resulting in biased models that may not accurately reflect the needs of underrepresented or vulnerable populations. For instance, Kiyasseh et al. (135) found that AI model could reliably assess surgical performance but exhibited bias. AI models trained primarily on data from affluent, homogeneous populations may not perform as well for patients from different racial, ethnic, or socioeconomic backgrounds, this raises serious concerns about equity in healthcare, as AI systems could inadvertently perpetuate disparities in diagnosis, treatment, and patient outcomes (136). For instance, Obermeyer et al. (54) found that an AI tool used in US hospitals to predict additional medical care disproportionately favored White patients over Black patients. Correcting this bias increased the percentage of Black patients receiving additional care from 17.7 to 46.5%, demonstrating the significant impact of algorithmic bias in clinical outcomes. To mitigate these biases, it is crucial to ensure that AI systems are designed to be fair and inclusive, with particular attention paid to the representation of minority groups in training datasets. Recent studies have proposed solutions such as algorithmic auditing tools, and collaborations between AI developers and ethicists to establish clear AI fairness protocols (137–139).

### 4.3 Privacy and ethical constraints in real-world healthcare

In practical terms, privacy concerns and ethical constraints can significantly impede the adoption of AI in healthcare. Healthcare institutions often face barriers related to data sharing due to patient privacy rights, institutional reluctance, and regulatory challenges (140). Even when data-sharing technologies such as FL or blockchain are employed, the complexity of synchronization and data sharing remains a significant hurdle (141). This creates challenges in developing AI models that require vast and varied datasets to enhance accuracy and ensure generalizability across diverse populations and clinical practices.

Moreover, ethical dilemmas arise when algorithms are used to make decisions that impact patient care without clear accountability. For example, if an AI system makes an incorrect diagnosis that leads to patient harm, determining responsibility becomes problematic. This issue is further complicated by the lack of clear governance frameworks for AI in healthcare. Currently, most healthcare systems do not have well-established processes to determine liability when AI systems make errors, which may hinder the widespread adoption of AI technologies (142, 143). Addressing these issues requires the development of clear, standardized governance structures that define the roles and responsibilities of healthcare practitioners, AI developers, and institutions (144–146).

Furthermore, addressing algorithmic bias is paramount to ensuring equitable AI performance across diverse patient populations. This requires the implementation of validated bias mitigation strategies—such as reweighting and adversarial debiasing during model training—and the rigorous application of fairness metrics (e.g., demographic parity, equalized odds) to quantify and monitor performance disparities across different demographic groups (147). Proactively auditing models for these metrics is essential to prevent the exacerbation of existing health disparities.

In addition to governance concerns, healthcare institutions may also face institutional resistance to adopting AI technologies. This resistance may stem from concerns about job displacement, lack of technological infrastructure, or trust in AI systems (148). To facilitate AI adoption, training programs for healthcare professionals are essential, especially to address concerns about the erosion of clinical skills when AI is over-relied upon (149). Additionally, healthcare providers must be educated on how AI can enhance rather than replace their clinical expertise, fostering trust in AI’s role in improving patient care (150). Institutional commitment to AI integration is key, and this requires substantial investments in both training and infrastructure development (151).

### 4.4 Limitations and unintended consequences of AI in healthcare

While AI holds immense potential to transform healthcare, it is essential to acknowledge its limitations and the unintended consequences that may arise from its deployment. Algorithmic bias, as discussed in Section 5.1, remains a significant challenge in clinical settings. AI systems trained on biased datasets can lead to unequal outcomes for different demographic groups, such as patients from diverse racial, ethnic, or socioeconomic backgrounds. Recent studies have proposed methods such as data diversification and fairness auditing to mitigate these biases (152, 153).

In clinical practice, AI-driven diagnostic tools may fail to generalize in real-world settings due to variations in patient populations, data quality, and clinical workflows (154). Furthermore, over-reliance on AI systems can result in the erosion of clinical skills among healthcare professionals, who may become overly dependent on AI recommendations (155). AI’s failure to account for real-world complexities also highlights the need for continuous validation and human oversight in healthcare AI deployment.

Regulatory challenges further complicate the integration of AI in healthcare. Different regions have varying regulations governing AI use in clinical settings, creating complexities for multinational healthcare systems. Issues such as the lack of standardization, long approval timelines, and concerns over data privacy and security hinder the widespread adoption of AI technologies in clinical practices (156). Researchers have proposed solutions such as the development of international AI regulatory frameworks and the establishment of clear guidelines for AI certification and accountability (157, 158).

Ethical concerns, particularly regarding data privacy and patient consent, must also be addressed. AI systems often require access to sensitive patient data, and it is crucial to ensure that patient consent is obtained transparently and that their privacy is safeguarded (159, 160). The implementation of XAI is one approach to improving transparency and trust, as it can help healthcare professionals better understand AI-driven decisions and incorporate them into their clinical practice (161).

Finally, there are practical barriers to AI adoption, including insufficient technological infrastructure, lack of training among healthcare practitioners, and institutional resistance to adopting AI technologies (162). Many healthcare systems, particularly in low-resource settings, may not have the necessary infrastructure to support AI tools, such as high-performance computing or sufficient data storage capacity (62). In addition, healthcare practitioners need appropriate training to effectively use AI systems, and they must trust AI’s potential to improve patient care (163). Overcoming these barriers requires both institutional commitment to technology adoption and comprehensive training programs for clinicians.

### 4.5 Future directions in ethical AI implementation

Looking forward, the development of robust ethical governance frameworks is essential to address the multifaceted challenges posed by AI in healthcare. These frameworks must prioritize patient consent, data security, algorithmic transparency, and fairness to build trust and ensure equitable healthcare delivery. Institutions should adopt policies that not only harness the benefits of AI but also mitigate potential risks, ensuring that AI technologies are deployed responsibly and ethically (164). A comprehensive approach is essential, encompassing secure data-sharing methods, enhanced transparency in algorithmic decision-making, and the establishment of clear ethical guidelines for AI deployment in healthcare (165).

Recent international initiatives have underscored the importance of coherent global AI governance frameworks in healthcare. The U.S. Food and Drug Administration (FDA) has proposed the Total Product Lifecycle (TPLC) regulatory approach for AI/ML-based medical devices, emphasizing continuous monitoring and adaptive learning within a controlled framework (166). Similarly, the European Union’s AI Act classifies AI systems based on risk categories, requiring transparency, human oversight, and post-market audits for high-risk applications in healthcare (167). The World Health Organization (WHO) has also introduced its “Ethics and Governance of Artificial Intelligence for Health” guidance, outlining six key principles—autonomy, safety, transparency, accountability, inclusiveness, and sustainability—to guide ethical AI use globally (168). Incorporating these frameworks can promote international harmonization of AI governance and ensure consistent accountability across healthcare systems.

In addition to global regulation, enhancing data accountability and clinical oversight remains critical. Healthcare institutions should adopt auditable data governance mechanisms, including version-controlled data pipelines, algorithmic logging, and external audit trails to ensure traceability of AI-driven decisions (133, 169). These mechanisms are the foundation for the practical, long-term maintenance of AI systems, enabling continuous monitoring for model drift—such as performance degradation due to shifts in clinical practice or patient demographics—and triggering the need for timely model recalibration or retraining (170). Transparent auditing not only improves data integrity but also fosters public trust. Furthermore, clinician oversight should be institutionalized by establishing AI oversight committees that review and validate high-impact AI recommendations, ensuring that human judgment remains central to clinical decision-making (171).

The emerging framework of Symbiotic AI (SAI) provides a promising approach to ethical AI implementation. SAI emphasizes a collaborative relationship between AI systems and human users, fostering mutual understanding and trust. In healthcare, SAI addresses critical ethical challenges such as data privacy, algorithmic transparency, and fairness by adopting a human-centered approach to AI design (172). For instance, SAI frameworks integrate XAI techniques to enhance the interpretability of AI decision-making for healthcare professionals, thereby mitigating the “black box” problem (173). These approaches make AI decisions more understandable and transparent, facilitating better integration into clinical workflows.

To clarify its conceptual boundaries, SAI should be distinguished from related frameworks such as Explainable AI (XAI) and human–AI teaming. While XAI primarily focuses on interpretability and transparency, and human–AI teaming emphasizes cooperative task execution, SAI extends beyond both by promoting continuous bidirectional learning between humans and AI systems (174–176). In operational terms, SAI functions as a mutually adaptive system: clinicians refine their decisions based on AI insights, while AI models dynamically learn from clinician feedback and contextual data (175, 177). Critically, this feedback loop should be expanded to include patients, ensuring that their experiences, preferences, and reported outcomes inform both clinical use and the iterative refinement of AI tools. This iterative process ensures that AI remains aligned with ethical standards, clinical reasoning, patient values, and human values.

Existing studies have demonstrated both successes and challenges in the deployment of SAI systems in healthcare. For example, Tschandl et al. (178) found that SAI frameworks incorporating XAI techniques significantly improved clinician trust and understanding of AI-driven diagnostic recommendations. However, Mollura et al. (179) reported difficulties in scaling SAI systems due to computational cost and the need for extensive data storage, especially in low-resource settings. In such environments, the computational demands of SAI models could limit their accessibility and effectiveness.

Furthermore, SAI promotes the use of FL and secure data-sharing protocols, ensuring patient privacy while facilitating cross-institutional collaboration (180). FL allows for collaborative model training without sharing sensitive patient data, which is particularly valuable in maintaining privacy across healthcare institutions with varying data access policies.

To address these challenges, lightweight AI alternatives are being explored to reduce the computational burden. For example, model pruning, quantization, and edge computing offer potential solutions for implementing SAI in low-resource environments (181). These lightweight models can achieve performance comparable to more computationally intensive models while reducing the need for high-performance computing infrastructure.

By focusing on the symbiotic relationship between AI and humans, SAI provides a robust framework for ethical AI deployment in healthcare. AI systems must be designed to align with human values and societal needs, ensuring that technological advancements contribute to ethical and equitable healthcare solutions.

## 5 Conclusion and implications for public health

This narrative review has synthesized evidence demonstrating the transformative role of AI across the precision medicine continuum, from diagnosis and treatment personalization to drug discovery and continuous health monitoring. These advancements signal a pivotal shift toward more proactive, data-driven healthcare.

However, the translation of this potential into widespread clinical practice is contingent upon addressing significant challenges. These include the need for more robust, multi-center clinical validation to solidify the evidence base, and the critical imperative to overcome issues of data privacy, algorithmic fairness, and - most importantly - model generalizability across diverse populations and healthcare settings. Tackling these challenges is essential to ensure that the benefits of AI do not exacerbate existing health disparities but rather contribute to greater health equity.

The integration of AI into precision medicine thus holds profound implications for public health. AI can empower a shift from reactive treatment to population-wide preventive health by enabling targeted screening and early intervention strategies, potentially reducing the long-term burden of disease. To realize this vision, future efforts must focus on developing AI models with inherent fairness and robustness, fostered through diverse data collection and collaborative frameworks like federated learning. Concurrently, the establishment of supportive policy and ethical guidelines is crucial to steward the equitable and trustworthy deployment of these powerful technologies, ensuring they serve the goal of improving health for all.
